# Carcinome épidermoïde sur lichen scléro-atrophique vulvaire: Epidermoid carcinoma associated with atrophic vulvar lichen sclerosus

**DOI:** 10.11604/pamj.2018.29.2.14414

**Published:** 2018-01-02

**Authors:** Youssef Zemmez, Mohammed Boui

**Affiliations:** 1 Service de Dermatologie, Hopital Militaire Mohamed V, Rabat, Maroc

**Keywords:** Lichen scléro-atrophique, carcinome épidermoïde, rurit vulvaire, Atrophic vulvar lichen sclerosu, epidermoid carcinoma, vulvar pruritis

## Abstract

We here report the case of a 58-year old female patient with no particular past medical history, presenting with a 5-year history of vulvar pruritis, drought and vulvodynia at the Department of Dermatology. Clinical examination showed diffuse porcelaine lesions at the level of the vulva, with a yellowish hemispheric nodular lesion measuring 1 cm in diameter on the whitish plates and some excoriated lesions due to scratching. A skin biopsy was performed at the level of the whitish lesions and at the level of the nodular lesion. Histological examination suggested epidermoid carcinoma for the budding lesion and atrophic vulvar lichen sclerosus for the porcelaine lesions. Surgical treatment was recommended associated with radiation therapy. This study aims to highlight the risk for atrophic vulvar lichen sclerosu degeneration.

## Image en médecine

Patiente de 58 ans, sans antécédent pathologique, consultant en dermatologie pour prurit vulvaire, sécheresse et vulvodynie évoluant depuis 5 ans. L'examen clinique a révélé des lésions diffuses porcelainées au niveau de la vulve, avec une lésion nodulaire hémisphérique jaunâtre mesurant 1 cm de diamètre sur les plaques blanchâtres, et des lésions excoriées liées au grattage. Une biopsie cutanée a été réalisée au niveau des lésions blanchâtres et au niveau de la lésion nodulaire. L'histologie était en faveur du carcinome épidermoïde pour la lésion bourgeonnante et en faveur du lichen scléro-atrophique (LSA) vulvaire pour les lésions de porcelaine. Un traitement chirurgical a été recommandé associé à la radiothérapie. L'intérêt de cette observation est de rappeler le risque de dégénérescence du lichen scléro-atrophique génital.

**Figure 1:**
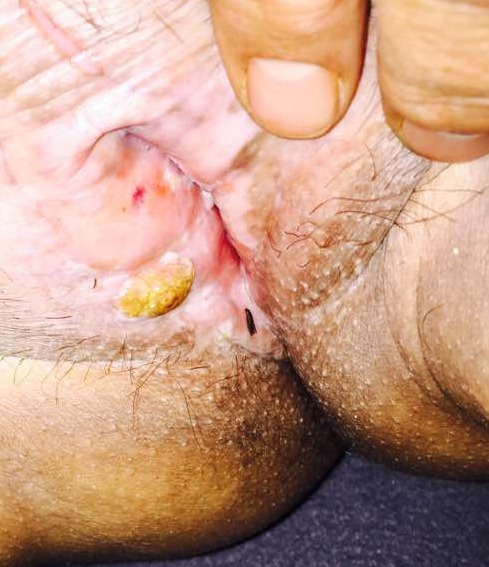
carcinome épidermoïde développé sur le lichen scléro-atrophique vulvaire

